# Inter- and Intrarater Reliability of Modified Lateral Scapular Slide Test in Healthy Athletic Men

**DOI:** 10.1155/2014/384149

**Published:** 2014-05-13

**Authors:** Azadeh Shadmehr, Mohammad Hassan Azarsa, Shohreh Jalaie

**Affiliations:** Enghelab Avenue, Pitch-e-shemiran, Tehran 11489-65141, Iran

## Abstract

*Objective*. The reliability of lateral scapular slide test (LSST) at 90 degrees of abduction is controversial; therefore, in order to achieve more reliability it may be necessary to make changes in this particular position. *Methods*. Modified lateral scapular slide test (MLSST) was done on thirty male basketball players with two examiners in one session and for the retest with one examiner in the next week. The test was done in 7 positions: arm relaxed at the side (P1), 90 degrees of abduction (P2), 90 degrees of scaption without having a weight in hands (P3), 90 degrees of scaption with having 3 different weights (1, 2, and 4 kg) in hands (P4, P5, and P6, resp.), and 180 degrees of scaption without having a weight in hands (P7). *Results*. In P1 and P6, the ICC scores indicated the highest level of intrarater reliability. In P2, the ICC scores showed a fair level of intrarater reliability, as the minimum reliability. The maximum and minimum interrater reliability were P1 and P4, respectively. *Conclusion*. Scaption with loading, as a functional position in the overhead athletes, is a reliable positioning and may be replaced with the third position of the traditional LSST.

## 1. Introduction

Alteration of scapular position during shoulder motions is commonly associated with injuries that create clinical dysfunction of the shoulder. In overhead sports in which demands placed on the shoulder are extremely high, abnormal scapular kinematics is more commonly involved [1].

The lateral scapular slide test (LSST) developed by Kibler [2] is a more available and clinical method of examining the scapular positioning; therefore, further efforts to improve the reliability of this test are more valuable and cause the test to be more clinical.

The reliability of LSST has been examined in many previous studies but the results were not satisfying and have shown controversial results at 90 degrees of abduction, especially [3–7]. Shadmehr et al. reported poor ICC scores (0.63) in this position for interrater reliability in patients with different shoulder dysfunctions [5]. On the other hand, da Costa et al. showed that, at 90 degrees of elevation in scapular plane, interrater reliability was fair (ICC = 0.74) and intrarater reliability was good (ICC = 0.85) [6]. Struyf et al. determined poor interrater reliability for 90 degrees of abduction with 1 kg loading (ICC = 0.63) in musicians [7].

With respect to the fair reliability in functional position of scaption, it seems that, with adding the loading to this position, its functional role is better determined and this modification may lead to more satisfying results than Struyf's study that added the loading to the abduction position. Scapular positioning is hypothesized to bear a direct relationship with muscle performance in basketball players who do overhead throwing. Additionally, kinematics alternations in scapular motion have been linked to shoulder pathologies [1]. As a result, basketball players are suitable for evaluating the effects of loading on the scapular kinematics.

Therefore, the main goal of the present study was to determine the reliability of LSST in scapular plane with various loadings in basketball players.

## 2. Materials and Methods

This was a cross-sectional, prospective, and repeated measures study of thirty athletes with two examiners in two sessions. Thirty healthy male basketball players, between 20 and 31 years old, were recruited from three basketball teams. Prior to participating in this study, all athletes signed an informed consent form. This study was approved by the ethics committee of Tehran University of Medical Sciences (TUMS).

The inclusion criteria for the participants were the ability to actively perform 90 and 180 degrees of scaption, abduction, and full internal rotation of the shoulder, the age of 20–40 years, and a minimum of 2 years of sports experiences or 3 to 4 exercise sessions per week in basketball. Athletes were excluded if they had the following problems: previous shoulder surgery, overuse injuries, a history of systemic diseases, and neuromuscular dysfunctions.

A digital Vernier caliper with an accuracy of 0.01 mm (Mitotuyo Company, Japan), a goniometer with extendable arm (Lafayette Instrument Company, USA), and 1, 2, and 4 kg dumbbell-like weights were used in these experiments. Demographic information including age, mass, height, and body mass index (BMI) was measured and related questions were answered. To determine the scaption plane, the basketball players stood the front of the wall corner on a reference plate with signs for foot stands and a reference line on it. To determine the 90 and 180 degrees of shoulder abduction, we set different parts of a goniometer as follows: a fixed extendable arm was set along with external body line; shaft of the goniometer was under the external part of acromion with a distance of 2 cm and the mobile arm parallel to athletes' arm axis in 90 or 180 degrees. To measure the scaption angle, we set the fixed goniometer arm on the body axis in frontal plane and we put the axis of goniometer on the acromion tip and the mobile arm parallel to athlete arm axis [8]. Since there may be some differences in participant's upper extremity length, we extended the extendable mobile arm of goniometer up the wall and marked crossing points by markers on it. This was done at 90 and 180 degrees of scaption for both upper extremities separately. All athletes were asked to keep upper extremities compatible with these markers. These markers were positioned on the wall in a way in which they made 40 degrees with frontal body plane to guide athletes along with scapula plane [6].

**Figure 1:**
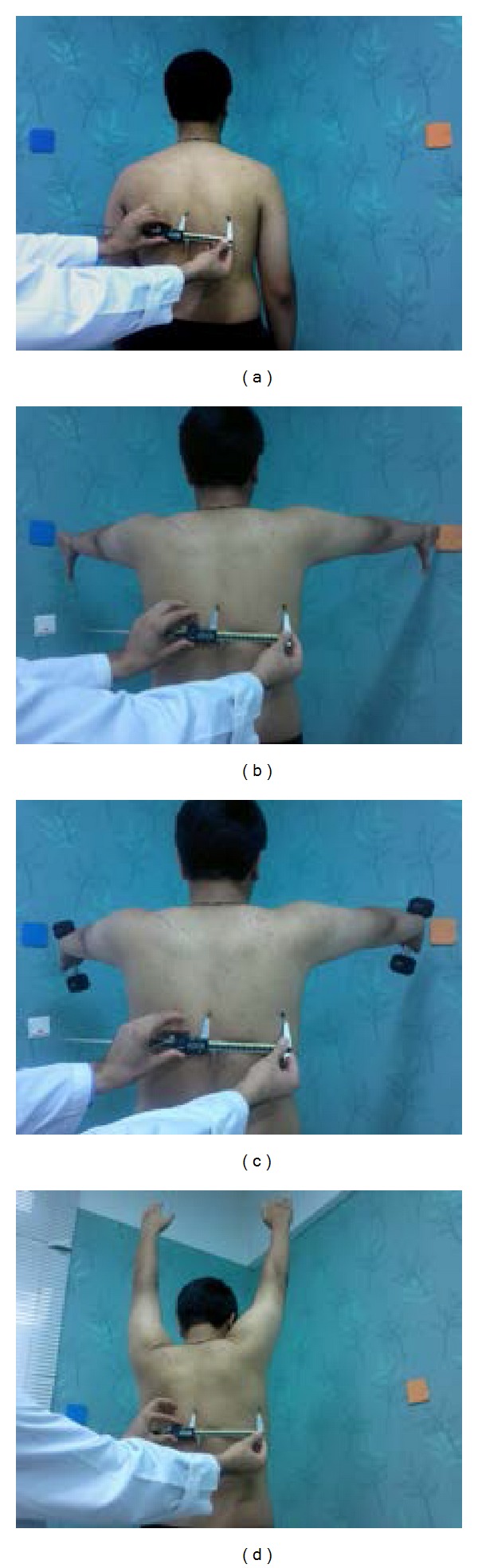
The measurement of the distance between the spinous process of T7 and inferior angle of scapula in (a) neutral position, (b) unloaded scaption, (c) loaded scaption, and (d) full scaption.

In the first position of the test (P1), the upper limbs were hanging beside the body and the examiner measured the least linear distance between T7 spinous process and inferior angle of scapula in 0.01 mm order using digital caliper (Mitutoyo Corporation, Japan) (Figure 1(a)). In the second position of the test (P2), the upper limbs were at 90 degrees of abduction with internal rotation of arms and the examiner found T7 spinous process and scapula inferior angle by touching; then, he measured the least linear distance between them using caliper in both sides. In the third position of the test (P3), the athlete was asked to keep his upper limbs at 90 degrees of scaption with internal rotation of shoulder (along with mentioned markers) without having a weight in his hands. Then 1, 2, and 4 kg dumbbell-like weights were given to athletes in the fourth (P4), fifth (P5), and sixth (P6) positions of the test, and they were asked to keep their upper limbs at 90 degrees of scaption with internal rotation of shoulder (along with mentioned markers) while keeping weights. In these positions the examiner measured the mentioned distance again (Figures 1(b) and 1(c)). In the seventh position of the test (P7), the athletes were asked to keep their trunk fixed and move up their arms along with markers put on the wall, without having weights in hands and as far as possible. In this condition the examiner measured the mentioned scapular distances using caliper (Figure 1(d)).

All of the above measurements were done by two randomly selected expert examiners with M.S. degree in 5-minute intervals (interrater). Before beginning the tests, examiners were qualified to find the landmarks and use caliper in measurements in sufficient time and in sports club. There was a 30-second break between two consecutive tests. Each examiner separately recorded the results in separate sheets without talking to other examiners about the test and its results. One of the examiners repeated the tests after one week exactly in the previous test times (intrarater).

Intraclass correlation coefficient (ICC) ([2,1] two-way random effects model) and 95% confidence interval (95% CI) for the ICC were used to analyze the reliability of MLSST. A 1-sample Kolmogorov-Smirnov goodness-of-fit test was done to determine normal distribution (*P* > 0.05; data not shown).

Standard error of measurement (SEM) equals the square root of the mean square of the error [9]. The ICCs were classified as follows: <0.69, poor correlation; 0.70–0.79, fair correlation; 0.80–0.89, good correlation; 0.90–1.00, high correlation [5]. All data were analyzed using SPSS version 19.

## 3. Results

In the present study, inter- and intrarater reliabilities of MLSST were determined in healthy basketball players.  Table 1 shows the demographic characteristics of the participants involved in this study.

Intrarater and interrater ICCs (single and average measures), 95% CI, and SEM are presented in Table 2. In P1 and P6 positions, the ICC scores indicated a high level of reliability for intrarater (0.94 and 0.90, resp.), as the maximum reliability. In P2 position, the ICC scores showed a fair level of reliability for intrarater (0.79), as the minimum reliability. The maximum and minimum interrater reliability were P1 (0.77) and P4 (0.54), respectively.

The highest and the lowest intrarater SEM were 0.74 cm and 0.32 cm for P2 and P1 positions, respectively. The results showed that the amount of errors in measurements of the two examiners (interrater) was higher than intrarater. The lowest error belonged to P1 (0.71 cm) and the highest amount of errors happened in P2 (1.08 cm).

## 4. Discussion

The purpose of this study was to evaluate the reliability of the MLSST in healthy overhead sportsmen. Our findings showed that the first position (P1) had the highest level of intrarater reliability. Previous studies confirmed our results [5–7]. Since in this position it is easy to touch inferior angle of scapula, furthermore, scapula remains in a static position and therefore it is easier to determine its location.

We found that, at 90 degrees of scaption (P3), there is good intrarater and fair interrater reliability. da Costa et al. in similar raters showed results similar to our study's [6]. The above findings may be due to the fact that scaption is the functional and true physiological movement of the shoulder abduction [10]. In this plane the glenohumeral capsule is not twisted and therefore the humeral movement is less restricted compared to the frontal plane [11]. As a result, in most of daily activities and sports, scaption is a dominant and comfortable position.

There is a natural scapulothoracic rhythm and muscle timing between glenohumeral abduction and scapulothoracic upward rotation accompanied by 20 degrees of scapular posterior tilt [12]. Chu et al. also showed that the range of posterior tilt of scapula is lower in the scaption position than in the abduction position [12]. By increasing the posterior tilt of the scapula, its inferior angle moves closer to the thorax and obtains a deeper position. Therefore, it is more difficult to touch the inferior angle of the scapula as a key point of LSST. It seems easier to touch the inferior angle of the scapula in scaption and this may be a reason for improving the reliability in this position.

In the second modification, we asked athletes to handle 1, 2, and 4 kg loads during 90 degrees of scaption. Our study showed, that by increasing the loads (1, 2, and 4 kg), to unloaded scaption position (ICC = 0.82), the ICC scores for intrarater reliability increased as well (0.86, 0.89, and 0.90, resp.). Our participants were basketball players whose dominant task is throwing a heavy ball, so with applying verified loads in scaption we could perform the LSST in a more functional position for these athletes. We believe that applying the loads in scaption recruits more motor units from stabilizing muscles of the scapula and leads to a higher coordination in surrounding muscles that have a major contributing role in the scapular mobility and stability.

Struyf et al. in their study applied 1 kg load to the healthy musicians at 90 degrees of abduction and showed poor reliability with this modification, which is in contrast with our results [7]. It can be attributed to different sampling, because the overhead players may tolerate applied loads in a more stable position and hence show more reliability in the test compared to the musicians.

Good reliability for intrarater in P7 showed that functional conditions can be very useful for musculoskeletal assessments. In this position, inferior angle of scapula moves to lateral position more than other positions and it has better capability to palpate in the inferior portion of the axilla. Thus, it is not unexpected that this position has good reliability. In addition, it is easier to hold the status relative to scaption (with and without weights) and abduction position for the athletes. Therefore, it causes minimal SEM in this position (SEM = 0.38 cm). Unlike the good reliability for intrarater in P7, fair interrater reliability (ICC = 0.71) shows that in above position the agreement between the raters is low.

Our results determined that the interrater reliability has lower scores compared to the intrarater reliability in all positions. Regarding the fact that the LSST includes two objective and subjective parts, it is noticeable that the results of the test are strongly dependent on the rater's experience and accuracy of bony landmarks determination. It may explain the lower scores of interrater reliability of the LSST. It is recommended in intermittent clinical settings to conduct the test by one examiner.

The interrater standard error of measurement (SEM), which indicates absolute reliability, was lower in this study in comparison with da Costa et al.'s study [6]. Better precision of caliper than palpation meter may explain apparent improvement in reliability. In the present study we showed a descending trend of SEM from P2 to P6 positions. This means that, by increasing the load, the error of measurement was decreased. On the other hand, it seems that SEM may be affected by samples characteristics. In the present study all participants (*n* = 30) were male with the same sport activity, while in Costa's study, the participants were from both genders (15 males and 15 females).

In this study, each position was measured only once by each of the raters and then they were recorded in questionnaires; therefore, we reported and analyzed single measures of lower and upper bound of 95% CI for ICCs limits. Table 2 shows that an average of several measurements improves the final reliability results and can be used in clinical application.

In this study for intrarater reliability there was one-week interval between the two tests which resolved memory effect in raters. A week is a good interval to avoid significant changes in shoulder posture of athletes while it has no significant clinical effect on the tests.

With respect to the limitations of this study, we can refer to the fact that a 30-minute session was held to explain the test condition and procedures to raters while in other studies such as McKenna et al.'s [13] the training session lasted for 4.5 hours and in Nijs et al.'s [14] it was 2 hours. Although both raters in our study had adequate experience in traditional test, longer sessions for familiarization of the raters could improve interrater reliability results.

Contradictory information about the effect of prior experience of examiners has been reported. For example, in Odom et al.'s study, despite a 4- to 7-year clinical experience of examiners, the reliability of the test was poor [15]. It is in contrast with Nijs et al.'s study that junior examiners obtained high interrater reliability [14].

## 5. Conclusion

In general, this study showed that applying the loads in scaption position of the LSST may improve the reliability of the test in sport men. Scaption with loading, as a functional position in the overhead athletes, is a reliable positioning and may be replaced with the third position of the traditional LSST. However, this study was a pilot and preliminary research on healthy athletes, and it investigated the effect of various loadings on MLSST reliability in them and it is necessary to do complementary studies for the results to be useful for patients. Also, future studies with different subjects may benefit from loading in scaption. It is recommended that these positions be investigated in another athletic group using weights (especially with 2 and 4 kg weights) to engage more muscles.

## Figures and Tables

**Table 1:** Mean, standard deviation (SD), and range of demographic data of participants (*n* = 30).

Demographic data	Mean (SD)	Range
Age (years)	22.53 (3.72)	20–31
Height (cm)	187.33 (9.81)	170–210
Mass (kg)	84.17 (16.29)	54–130
BMI* (kg/m^2^)	23.83 (3.04)	18.68–30.26
Sports experience (years)	8 (5.5)	2–20

*BMI: body mass index.

**Table 2:** Mean and standard deviation (SD) of measurement scores, intraclass correlation coefficient [ICC; 95% confidence interval (CI)], and standard error of measurement (SEM) for intrarater and interrater reliability.

Position	Intrarater reliability			Interrater reliability		
	Single measurement ICC (95% CI)	Average measurement ICC (95% CI)	SEM (cm)	Single measurement ICC (95% CI)	Average measurement ICC (95% CI)	SEM (cm)
P1	0.94 (0.90–0.96)	0.97 (0.95–0.98)	0.32	0.77 (0.65–0.86)	0.87 (0.79–0.92)	0.71
P2	0.79 (0.67–0.87)	0.88 (0.80–0.93)	0.74	0.63 (0.45–0.76)	0.77 (0.62–0.86)	1.08
P3	0.82 (0.71–0.89)	0.90 (0.83–0.94)	0.59	0.73 (0.59–0.83)	0.85 (0.74–0.91)	0.82
P4	0.86 (0.78–0.91)	0.92 (0.87–0.95)	0.45	0.54 (0.33–0.69)	0.70 (0.50–0.82)	0.97
P5	0.89 (0.82–0.93)	0.94 (0.90–0.96)	0.45	0.67 (0.50–0.79)	0.80 (0.67–0.88)	0.78
P6	0.90 (0.84–0.94)	0.95 (0.91–0.97)	0.41	0.64 (0.47–0.77)	0.78 (0.64–0.87)	0.78
P7	0.87 (0.78–0.92)	0.93 (0.88–0.96)	0.37	0.56 (0.35–0.71)	0.71 (0.52–0.83)	0.91

Note: arm is relaxed at the side (P1), 90 degrees of abduction (P2), 90 degrees of scaption without having a weight in hands (P3), 90 degrees of scaption with having 3 different weights (1, 2, and 4 kg) in hands (P4, P5, and P6, resp.), and 180 degrees of scaption without having a weight in hands (P7).
